# Spatial modeling of cutaneous leishmaniasis in the Andean region of Colombia

**DOI:** 10.1590/0074-02760160074

**Published:** 2016-06-27

**Authors:** Mauricio Pérez-Flórez, Clara Beatriz Ocampo, Carlos Valderrama-Ardila, Neal Alexander

**Affiliations:** 1 Centro Internacional de Entrenamiento e Investigaciones Médicas, Cali, Colombia; 2 Pontificia Universidad Javeriana, Cali, Colombia; 3 Universidad Icesi, Departamento de Ciencias Biológicas, Cali, Colombia

**Keywords:** cutaneous leishmaniasis, environmental risk factors, Colombian Andean region, spatial analysis

## Abstract

The objective of this research was to identify environmental risk factors for cutaneous leishmaniasis (CL) in Colombia and map high-risk municipalities. The study area was the Colombian Andean region, comprising 715 rural and urban municipalities. We used 10 years of CL surveillance: 2000-2009. We used spatial-temporal analysis - conditional autoregressive Poisson random effects modelling - in a Bayesian framework to model the dependence of municipality-level incidence on land use, climate, elevation and population density. Bivariable spatial analysis identified rainforests, forests and secondary vegetation, temperature, and annual precipitation as positively associated with CL incidence. By contrast, livestock agroecosystems and temperature seasonality were negatively associated. Multivariable analysis identified land use - rainforests and agro-livestock - and climate - temperature, rainfall and temperature seasonality - as best predictors of CL. We conclude that climate and land use can be used to identify areas at high risk of CL and that this approach is potentially applicable elsewhere in Latin America.

The World Health Organization (WHO) has classified leishmaniasis as a serious global public health problem, and as a neglected and re-emergent disease (WHO 2007). It comprises a group of diseases - cutaneous (CL), muco-cutaneous (ML) and visceral (VL) - caused by *Leishmania* parasites which, in the Americas, are transmitted by female *Lutzomyia* sandflies (Chaves & Pascual 2006). Like all vector-borne infectious diseases (VBD), leishmaniasis is highly sensitive to environmental factors (Weber 2010). Climate affects the biology and ecology of the triad parasite-vector-reservoir, and hence the disease’s spatial and temporal distribution (Ready 2010).

Vector distributions have been found to track environmental changes (Gebre-Michael et al. 2004, Michalsky et al. 2009), and WHO is promoting integration of new technological VBD monitoring tools, such as remote sensing and geographic information systems (GIS) (Bavia et al. 2005). Factors such as temperature, precipitation, vegetation cover, land use and natural phenomena such as El Niño or La Niña, have been associated with the occurrence of CL or VL, in patterns which depend on the region, ecological context and vector species (King et al. 2004, Bhunia et al. 2010, Valderrama-Ardila et al. 2010).

The global incidence of CL is estimated at 1.5 million new cases each year (OMS 2010). In the Americas the reported CL cases/year was 66,941 between 2003-2008 (Alvar et al. 2012). In Colombia the annual number of reported cases has increased from an average of approximately 6,500 in the 1990s; 14,000 in the 2000s; to 17,420 between 2005-2009, CL comprising more than 95% of these (INS 2010, Alvar et al. 2012). Major epidemics in Colombia have occurred in the Andean region (Pardo et al. 1999, 2006, Cárdenas et al. 2005, 2006, Flórez et al. 2006, Santamaria et al. 2006, Rodríguez-Barraquer et al. 2008), with *Lutzomyia* species from the *verrucarum* group being the dominant vectors (Bejarano et al. 2003).

The current study seeks to identify environmental factors associated with the incidence of CL in the Colombian Andes and hence map the risk at the level of municipality. This map is expected to serve as a support tool for surveillance of leishmaniasis in Colombia and contribute to reducing the impact of this disease.

## MATERIALS AND METHODS

**Fig.1:**
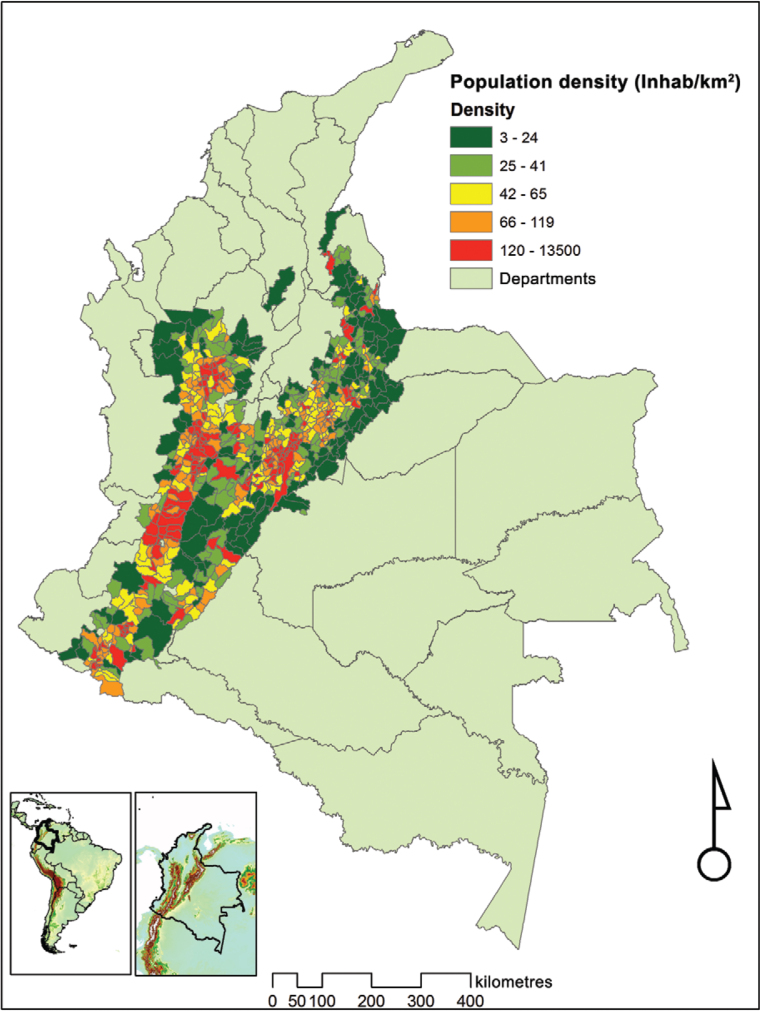
: map of the area of study (Andean region) showing population density (number of inhabitants per square kilometre).

*Population and study area* - The study area was the Colombian Andean region which comprises 24.5% of the country’s land surface area, or 278,600 km^2^ (Etter & van Wyngaarden 2000) and includes the western, central and eastern mountain ranges (cordilleras), and much of the Magdalena and Cauca river valleys (Fig. 1). More specifically, we followed the definition used by the Instituto Alexander von Humboldt (IAvH) by taking those parts of the country above 400 m (Rodríguez et al. 2006). This region is characterised by a mosaic of savanna ecological regions, tropical forests and wooded areas with diverse topography, climate and vegetation (King et al. 2004, Sandoval et al. 2006, Cárdenas et al. 2008). It also includes large areas devoted to agriculture as well as the country’s most populous cities.

The unit of analysis was the municipality because this is the finest level captured consistently by epidemiological surveillance in Colombia. Of 860 rural and urban municipalities overlapping the Andean region, i.e. lying at least partly above 400 m, the study included those 715 having more than 90% of their area within this region, i.e. altitude above 400 m. These 715 municipalities belong mainly (n = 696) to 13 Andean departments (states) (Boyacá, Cundinamarca, Antioquia, Santander, Nariño, Tolima, Valle del Cauca, Cauca, Huila, Norte de Santander, Caldas, Risaralda and Quindío), with the remaining 19 from six other departments (Casanare, Meta, Putumayo, Chocó, Bolivar and Cesar). According to the DANE (2005) national census, the population of these 715 municipalities was 27,416,481 (66.1% of national total), averaging approximately 38,000 each.

*Variables and sources* - *CL incidence* - The outcome of interest is the annual incidence rate of CL for the period 2000-2009. The numerator (annual number of CL cases by municipality) comes from the mandatory reporting system SIVIGILA, supplied by the Ministry of Health and Social Protection (Ministerio de Salud y Protección Social or MSPS) and are new and recurrent reported cases of CL. Data were considered good quality, and retained in the analysis, if they had information on municipality of origin and type of disease (CL, ML or VL). The denominator (number of people) at the middle of the study period was taken from the DANE (2005) national census or population projections. The latter were used when DANE reported partial information on the number of inhabitants due to access problems at the time of the census.

We use all available environmental variables. We consider 31 variables as potential environmental risk factors: 19 climatic, one topographic, one demographic and 10 on land use.

*Land use* - Land use was obtained from the Colombian Andes ecosystems map of IAvH which was made at a scale of 1:250,000 (IAvH 2005, Rodríguez et al. 2006) from interpretation of satellite images and thematic information for the year 2005. This map is in digital raster format (.img) of 32x32 metres but was manipulated in vector format. The 1:250,000 scale refers to the level of detail at which satellite images were interpreted. This map identifies 142 different ecosystems of the Colombian Andean region (112 natural and 30 modified) (Rodríguez et al. 2006), grouped in 10 categories of coverage for the current study: rainforests; dry forests; forest and secondary vegetation; shrub and xeric vegetation; *páramo* (alpine ecosystem above the tree line) and snow; coffee agroecosystems; mechanised crops (rice and sugarcane); livestock agroecosystems; unmechanised crops, such as fruit, vegetables and intercropping; others (urban areas, marshes, ponds, lakes, lagoons, rivers, beaches, wasteland, etc.).

*Climate* - Bioclimatic variables (Supplementary data) come from remote sensing systems available in the WorldClim database (http://www.worldclim.org). We used 19 climate surfaces (raster format at spatial resolution of 1 km) derived from monthly values of temperature (ºC) and precipitation (mm), e.g. annual average, precipitation or mean temperature in the coldest quarter, and similarly for the warmest, wettest and driest quarters (Hijmans et al. 2005).

*Elevation* - A raster image of a NASA digital elevation model was acquired in EROS Data Center (edc.usgs.gov), from the mission “Shuttle Radar Topography Mission - SRTM”, at a resolution of 3 arc-seconds (~90m).

*Population density* - Population density (Fig. 1), i.e. the number of inhabitants per square kilometre, was obtained for each municipality, based on the DANE (2005) national census and a digital of map of the municipalities from IGAC (1:100.000).

*Information processing* - ArcMap 9.3 software with Spatial Analyst was used for analysis of shapefile and raster data, and for extracting numeric data from satellite images. It was used to overlap the map of municipalities with the ecosystem (ground coverage) map and the 19 bioclimatic and elevation layers. Then summary statistics were obtained for each municipality, such as percentage coverage with each ecosystem, and averages of climatic variables and elevation.

*Statistical analysis* - *Correlation analysis* - Collinearity between the variables, in terms of Spearman’s correlation coefficient (*r*), was used to guide data reduction.

*Spatial analysis* - We used a random-effects Poisson model, allowing spatial correlation via an intrinsic conditional autoregressive (CAR) prior. The response variable (*Y*_*it*_) was the total number of CL cases in municipality *i* by year in 2000-2009 (*t =* 1,…,10), and independent variables (*x*_*i*_) were the selected land use and climatic factors, population density and year (Supplementary data). The model included a logarithmic link function and logarithm of population size as an offset, so that exponentiating the regression coefficients *a*_*i*_ estimates incidence rate ratios (IRRs), not standardised for age. These are reported with their respective Bayesian 95% credible intervals (95% CI_cred_). We chose this CAR model as it takes into account the lack of independence in the data - i.e. spatial autocorrelation - and uses it to improve estimates based on neighbor information (Gelfand & Vounatsou 2003, Heinen & Rengifo 2003, Ready 2008). We also included unstructured random effects (Thomas et al. 2004). For the annual average temperature, a quadratic relationship was considered because of the vector’s aversion to extreme hot or cold and predominance at intermediate altitudes (Pardo et al. 1999, Young & Duncan 1994).

Spatial analysis was conducted in a Bayesian framework using non-informative large-variance priors. The intercept (*a*_*0*_) was assigned an improper uniform prior distribution (Thomas et al. 2004). The other *a’*s were assigned normal priors distributions with mean zero and variance 10^5^. For the structured components (*b*_*i*_), a Gaussian CAR prior distribution was assumed, with contiguity as criterion of proximity between municipalities and a scalar argument *t*_*b*_ representing the precision parameter (inverse variance). For *h*_*i*_, normally distributed priors with zero mean and variance 1/*t*_*h*_ were assumed. For both *t*_*b*_ and *t*_*h*_, Gamma priors with parameters 0.5 and 0.0005 (mean 10^3^ and variance 2x10^6^) were assumed. In sensitivity analysis, Gamma priors with parameters 0.1 and 0.1 (mean 1 and variance 100) were used for *t*_*b*_ and *t*_*h*_.

Spatial analysis used the GeoBUGS v1.4.3 component of the WinBUGS v1.2 software. The map of municipalities was converted from shapefile to SPlus format using a program written by Ms Yue Cui, University of Minnesota (Cui 2006).

Models were fitted using Markov Chain Monte Carlo. In order to speed convergence in the estimation of the parameters and reduce autocorrelation, each independent variable was centered by subtracting its average (Thomas et al. 2004). Two chains were run with different starting values. Convergence was evaluated by monitoring parameters *a*_*0*_ *, a*_*k*_ *, a*_*i*_ *, t*_*b*_ and *t*_*h*_. After 50,000 ‘burn-in’ iterations, visual inspection of the trace plots showed stabilisation and overlap of the two chains, and on this basis we concluded that convergence had occurred. 50,000 additional iterations were run for estimation of parameters’ posterior distributions.

Spatial analysis consisted of two phases: bivariable, then multivariable analysis using stepwise-forward selection. The model started with the independent variable most associated with the outcome (on basis of size of *a*_*i*_). New variables (biologically important and statistically significant) were added until no more were statistically significant.

*Risk map* - The results of the multivariate model were used to estimate the expected number of cases for each year. For the last year of the study period, 2009, these expected numbers were used to estimate the corresponding rates for each municipality, which were mapped using ArcMap 9.3. This software was also used to generate choropleth maps of observed incidence rate of CL by municipality. Approval was obtained from the Institutional Ethics Committee on Human Research (CIEIH) of CIDEIM and the Institutional Ethics Committee for Human Research of Universidad del Valle. The Ministry of Health and Social Protection (MSPS) also approved the use of its epidemiological data.

## RESULTS

**Fig. 2:**
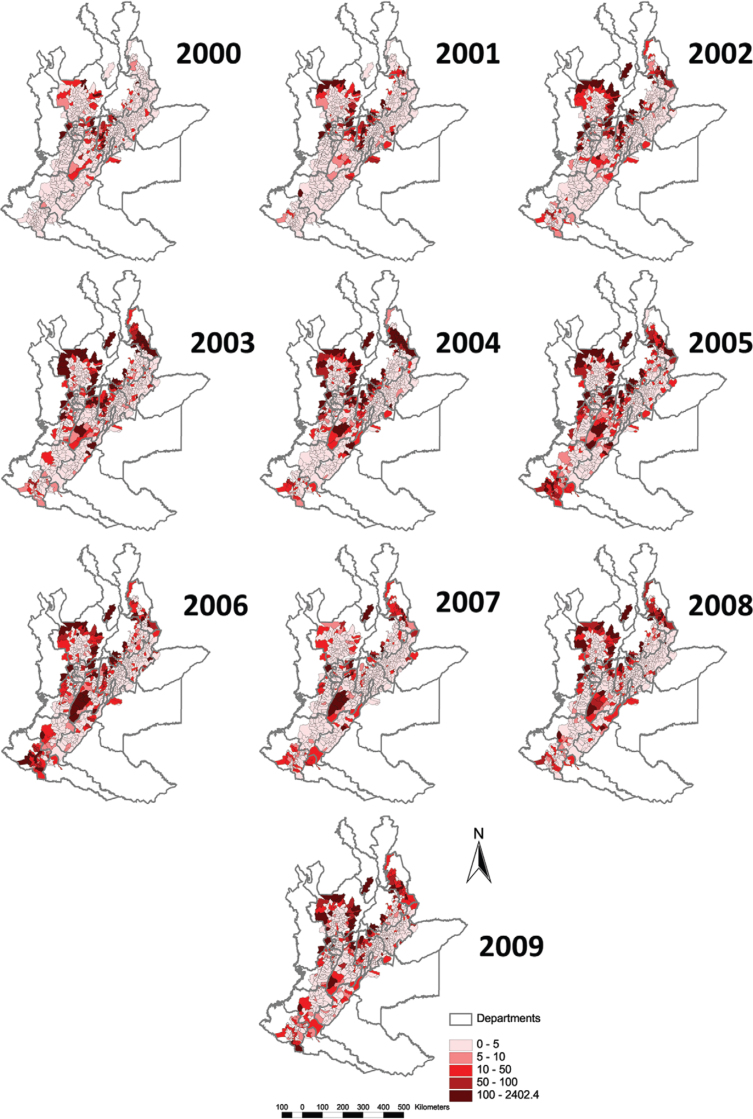
: observed incidence rate of cutaneous leishmaniasis in the Andean region of Colombia by year from 2000-2009 (per 100,000 inhabitants) (Source: Ministry of Health and Social Protection).

*Incidence* - A total of 90,413 cases of leishmaniasis (CL, ML, VL and unspecified) were reported in Colombia in the period 2000-2009, of which 85.4% (77,210/90,413) had the municipality recorded and only 2% were unspecified cases. Of these, 78.7% (60,794/77,210) are from the 860 municipalities that fully or partially overlap with the Andean region (above 400 m), with 38.8% (29,949/77,210) from the 715 municipalities in the main analysis on the basis of having more than 90% of their surface area in the region. We use 29,542 cases of CL in the Andean region giving an overall incidence rate of 107.8 cases (29,542/27,416,481) per 100,000 inhabitants for the ten-year period, or 10.8/100,000/year. We performed a temporal analysis of incidence rates per year (Fig. 2).

*Bivariable analysis* - Six temperature variables were strongly correlated (*r* > 0.7) with mean annual temperature and six precipitation variables with annual precipitation: these 12 were excluded from spatial analysis (Supplementary data). There was also a strong negative correlation between the mean annual temperature and elevation (r = -0.991), so the latter was also excluded, on the basis of temperature having a more direct effect on the vector.

In addition, *páramo* coverage was excluded due to its lack of contribution to transmission in Colombia, as was the ‘other’ category for being too heterogeneous, and (i) dry forest, (ii) shrubland and xeric vegetation, and (iii) technified crops for their rarity (0.03%, 0.6% and 1.7% respectively). Finally, several climatic variables were excluded on the basis of homogeneity over the study area, specifically average annual temperature diurnal range, isothermality (mean diurnal temperature range as a percentage of annual range) and annual temperature range, with coefficients of variation 7.7%, 5.5% and 9.0%, respectively. Ten variables showing low collinearity (all 45 distinct off-diagonal pairwise correlations < 0.7) were retained for spatial analysis (Table I).

**TABLE I:** Bivariable spatial analysis of factors associated with cutaneous leishmaniasis incidence in the Andean region of Colombia for 2000-2009

Independent variable	IRR	Change (%)	95% CI_Cred_^a^
Rainforests (%)	1.028*	2.8%	(2.0%; 3.7%)
Forest and secondary vegetation (%)	1.021*	2.1%	(0.6%; 3.6%)
Coffee agroecosystems in association (%)	1.003	0.3%	(-1.8%; 2.5%)
Livestock agroecosystems (%)	0.970*	-3.0%	(-4.1%; -1.9%)
Non-technified crops (%)	1.002	0.2%	(-0.7%; 1.2%)
Bio1: Annual mean temperature (°C)			
- Linear	1.155*	15.5%	(10.2%; 21.2%)
- Quadratic	0.985*	-1.5%	(-2.2%; -0.7%)
Bio4: Temperature seasonality (°C)	0.970*	-3.0%	(-5.9%; -0.1%)
Bio12: Annual precipitation (mm)	1.0016*	0.16%	(0.1%; 0.2%)
Bio15: Precipitation seasonality (%)	0.995	-0.5%	(-3.9%; 3.2%)
Population density (per inhabitant per km^2^)	1.001	0.1%	(-0.4%; 0.6%)

*a*: 95% CI_Cred_: 95% Bayesian credible interval; *: statistically significant (CI excludes 1); IRR: incidence rate ratio.

The first phase of spatial analysis used 10 independent variables and six were associated with CL incidence. The incidence showed an inverted U-shaped (quadratic) relationship with average annual temperature, however this significance disappeared in multivariable analysis.

CL incidence was positively associated with rainforest cover, with a 2.8% increase (95% CI_Cred_: 2.0-3.7%) per additional percent of coverage. It also increased by 2.1% (95% CI_Cred_: 0.6-3.6%) for each additional percent coverage with forest and secondary vegetation.

CL incidence was positively associated with annual rainfall, increasing 0.16% (95% CI_Cred_: 0.12-0.20%) per millimetre. By contrast, livestock agroecosystems and temperature seasonality (i.e. standard deviation of monthly temperature averages) showed a negative association with CL incidence, decreasing 3.0% (95% CI_Cred_: 1.9-4.1%) per percent coverage with livestock agroecosystems and 3.0% (95% CI_Cred_: 0.1-5.9%) per ºC of seasonal variation in temperature.

*Multivariable analysis* - In model 1 rainforest coverage was added to temperature and both retained statistically significance (Table II). Then annual precipitation and livestock agroecosystems coverage were added in turn (models 2 and 3). After we added temperature seasonality and all five variables were statistically significant (model 4). Finally, when the forest and secondary vegetation coverage was added, it lacked statistical significance, so model 4 was considered final.

**TABLE II:** Multivariable spatial analysis of factors associated with cutaneous leishmaniasis incidence in the Andean region of Colombia for 2000-2009

Model	IRR	95% CI_Cred_^a^
Model 1: Temperature + Rainforest coverage		
Bio1: Annual mean temperature (°C) - Linear	1.196*	(1.1350; 1.2633)
Quadratic	0.991*	(0.9827; 0.9983)
Rainforest (%)	1.034*	(1.0242; 1.0438)
Model 2: Temperature + Rainforests + Precipitation		
Bio1: Annual mean temperature (°C) - Linear	1.151*	(1.0924; 1.2115)
Quadratic	0.992	(0.9847; 1.0004)
Rainforests (%)	1.023*	(1.0123; 1.0321)
Bio12: Annual precipitation (mm)	1.001*	(1.0004; 1.0013)
Model 3: Temperature + Rainforests + Precipitation + Livestock agroecosystems		
Bio1: Annual mean temperature (°C) - Linear	1.147*	(1.0908; 1.2080)
Rainforests (%)	1.019*	(1.0088; 1.0314)
Bio12: Annual precipitation (mm)	1.001*	(1.0005; 1.0013)
Livestock agroecosystems (%)	0.988*	(0.9765; 0.9986)
Model 4: Temperature + Rainforests + Precipitation + Livestock agroecosystems + Temperature seasonality		
Bio1: Annual mean temperature (°C) - Linear	1.176*	(1.1158; 1.2450)
Rainforests (%)	1.017*	(1.0061; 1.0289)
Bio12: Annual precipitation (mm)	1.001*	(1.0004; 1.0014)
Livestock agroecosystems (%)	0.986*	(0.9753; 0.9977)
Temperature seasonality (°C)	0.942	(0.9152; 0.9673)

*a*: 95% CI_Cred_: 95% Bayesian credible interval; *: statistically significant (CI excludes 1); IRR: incidence rate ratio.

**Fig. 3:**
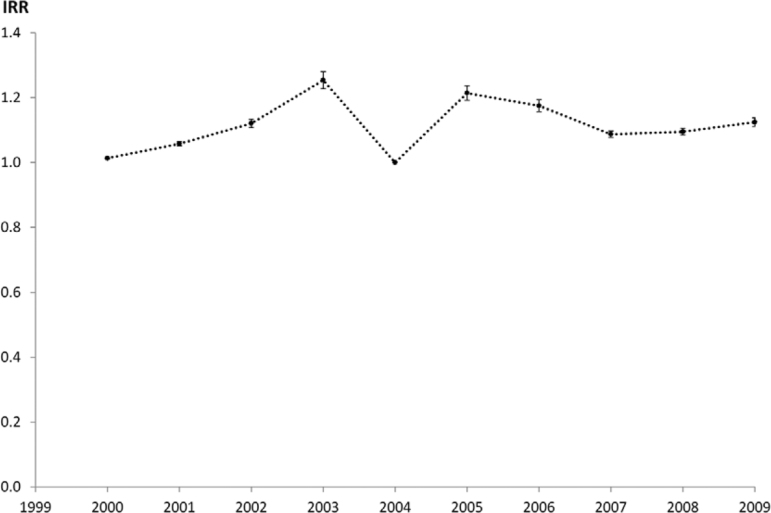
: incidence rate ratio over time, relative to 2004.

The temporal component of the analysis (Fig. 3) had as reference level the year 2004 (IRR = 1). CL incidence increased from 2000-2003, dropped sharply in 2004 before rebounding to another peak in 2005, then dropping back again.

**Fig. 4:**
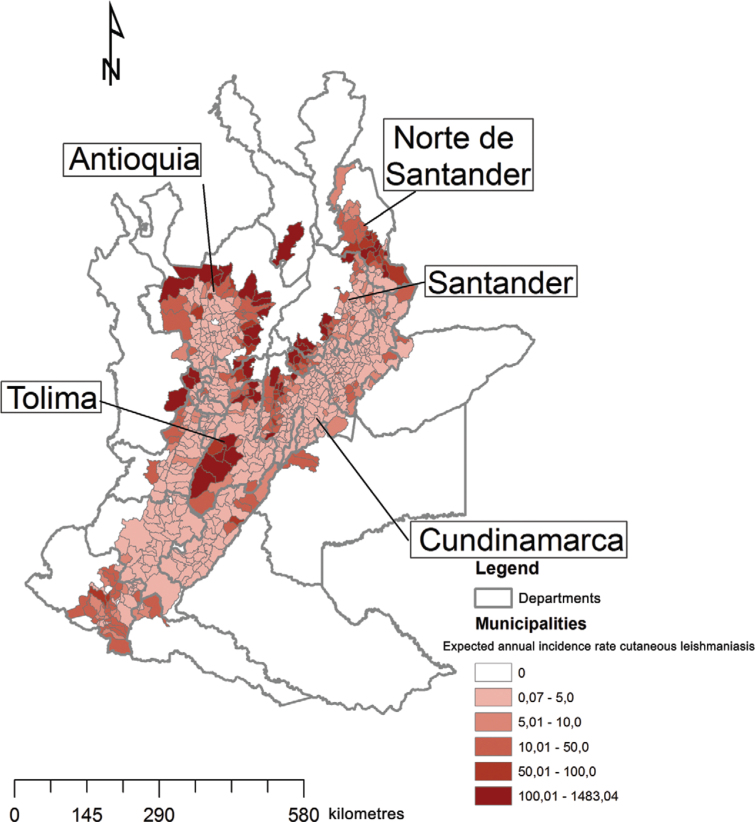
: risk map of expected incidence (per 100,000 inhabitants) in the Andean region of Colombia, by municipality, obtained from a predictive model based on environmental factors (rainforest coverage, coverage livestock agroecosystems, temperature, rainfall and temperature seasonality).

*Risk map* - Municipality-level risks were mapped from the expected annual incidence rates from model 4 (Fig. 4). It is noted that municipalities at highest risk (expected annual rate > 100 cases per 100,000 inhabitants) belong mainly to departments of Santander and Norte de Santander, Antioquia, Cundinamarca and Tolima.

## DISCUSSION

Leishmaniasis incidence in Colombia has increased over time and in spatial extent, suggesting a need for new surveillance tools (Pinzon et al. 2005). The current study used an “ecologic” design (analysis of aggregated data) to identify environmental factors associated with leishmaniasis incidence in the Colombian Andes.

*Incidence rate* - We restricted the study to the Andean region because the detailed IAvH ecosystem mapping was not available for other parts of Colombia. We found an overall incidence rate of 10.8/100,000/year in the Andean region. The deficit relative to the national rate (23.4) can be attributed to this study region containing the largest urban centres. The 715 municipalities lying at least 90% within this region contributed 38.8% of those cases whose location was recorded. The 145 municipalities partly overlapping the Andean region, but with at least of 10% of their area outside - so not included in the current study - contributed 30,845 cases, compared to 29,949 from 715 municipalities in the main analysis. Correspondingly, a high incidence is visible in Fig. 4 round the edges of the study region. Hence a surprisingly high proportion of cases originated from the fringes of the Andean region.

Most studies exploring environmental risk factors for CL have reported numbers of cases with no population denominator. The few calculations of incidence in such studies include 28.5 per 100,000 per year in the state of Bahia, Brazil (Franke et al. 2002) and 670 in Sidi-Bouzid, Tunisia (Salah et al. 2007), both much higher than that described in the current paper.

*Environmental factors associated with incidence of CL* - Multivariable spatial analysis found five variables associated with CL incidence. A positive association with rainforest cover was found, as by Valderrama-Ardila et al. (2010) in their smaller scale study in Chaparral (Tolima), although here it is more precisely estimated. We found a negative association between incidence and livestock agroecosystems. This does not seem to have been described previously although can be explained by transformation of natural landscape from forest into grasslands (Zeilhofer et al. 2008, Gil et al. 2010). Replacement of forests by anthropogenic vegetation like crops and pastures has led to destruction of the natural habitat of vectors and reservoirs, decreasing the risk of infection (WHO 2007, Zeilhofer et al. 2008, Gil et al. 2010).

We found a positive relationship between CL incidence and temperature. This is consistent with other studies in which temperature was associated with distribution of vectors (Thomson et al. 1999, Gebre-Michael et al. 2004, González et al. 2010) or human CL cases (Elnaiem et al. 2003, Chaves & Pascual 2006, Bhunia et al. 2010).

In this study annual rainfall was also positively associated with CL incidence which is consistent with most previous studies (Thomson et al. 1999, Elnaiem et al. 2003, Ready 2008, Bhunia et al. 2010). In some places, higher rainfall tends to increase vegetation density (Michalsky et al. 2009) and hence vector abundance (Ostfeld et al. 2005), at least at a coarse level (Githeko et al. 2000). Higher annual rainfall may be associated with habitats, such as forest coverage, which favor transmission (Kapos et al. 1997, Turton & Freiburger 1997). The negative association between temperature seasonality and incidence may be driven by the high seasonality in the southern fringe of the eastern cordillera, where it descends towards the eastern plains, conditions unfavorable to many phlebotomine species (Supplementary figure).

To our knowledge, this is the first spatial and temporal analysis of environmental risk factors for the Colombian Andean region. The map of King et al. (2004) used data from a single year (1994), with dichotomous response variable (presence or absence of at least one case reported in a municipality), and classical (non-spatial) logistic regression. A comparative visual analysis of the Andean region in these two risk maps does not seem to show a good agreement.

On the other hand, this does highlight a limitation of our study, namely that it modeled spatial-temporal variation in CL rate but not in climate and land use. For example, it cannot predict changes in incidence associated with inter-annual changes due to the El Niño climate phenomenon, as have been identified in northeastern Colombia (Cárdenas et al. 2006) or other causes. The temperature and rainfall data are from the WorldClim database which was constructed from data mainly from 1950-2000 and then interpolated to pixels of 1 km^2^. For the Andean region, uncertainty in these WorldClim estimates ranged from 0.2 to > 0.4ºC for temperature, and > 10 mm for precipitation (Hijmans et al. 2005). At a finer time scale, in the Andean zone of Colombia, periods of lower rainfall were inversely associated with abundance peaks of *Lutzomyia longiflocosa* and *L. columbiana* vectors from the *verrrucarum* group that are the dominant species in the Andean region (Cárdenas et al. 2006, Ferro et al. 2011).

“Ecologic” studies are more susceptible to bias than those based on individuals (Borja-Aburto 2000) and they often - as here - use secondary data with possible measurement errors. Their aggregate-level analysis also precludes control of confounding at finer levels (Borja-Aburto 2000, Rochlin et al. 2011). This study used several data sources. Epidemiological data supplied by the public health surveillance in Colombia can have several limitations. For example, missing municipality information allowed use of only 85% of the records. Under-reporting is recognised nationally (King et al. 2004) and internationally (Miranda et al. 2005, Ready 2008, INS 2010). Changes in notification protocol over a period of nine years, a misclassification of origin municipality and migration of populations, among others, may cause over - or under - estimation of the true incidence rate. Municipal-level data aggregation is quite coarse, but this is the finest level at which Colombian surveillance data are compiled. For each municipality we represented temperature, rainfall and elevation by a single value (average) ignoring intra-municipal variation in climate and topography, which may be important for CL (Valderrama-Ardila et al. 2010).

On the other hand, in terms of strengths, we were able to include land use at a fine spatial resolution (1:250,000) and incidence data from a long period (10 years). Integration of GIS data from remote sensing with spatial modeling and epidemiological data allowed identification of environmental risk factors and construction of the first municipality-level risk map for CL in the Colombian Andes.

In conclusion, in this spatially and temporally extensive study of climate and land use as potential risk factors, we found that the incidence of CL in the Andean region of Colombia is positively associated with mean temperature, rainforest coverage and precipitation, and negatively associated with livestock agroecosystems and temperature seasonality. The integration of epidemiological data, remote sensing images, GIS and statistical modeling can be used to identify areas at high risk of CL. This approach is potentially applicable elsewhere in Latin America to support vector borne disease surveillance systems.

## Figures and Tables

**Figure:**
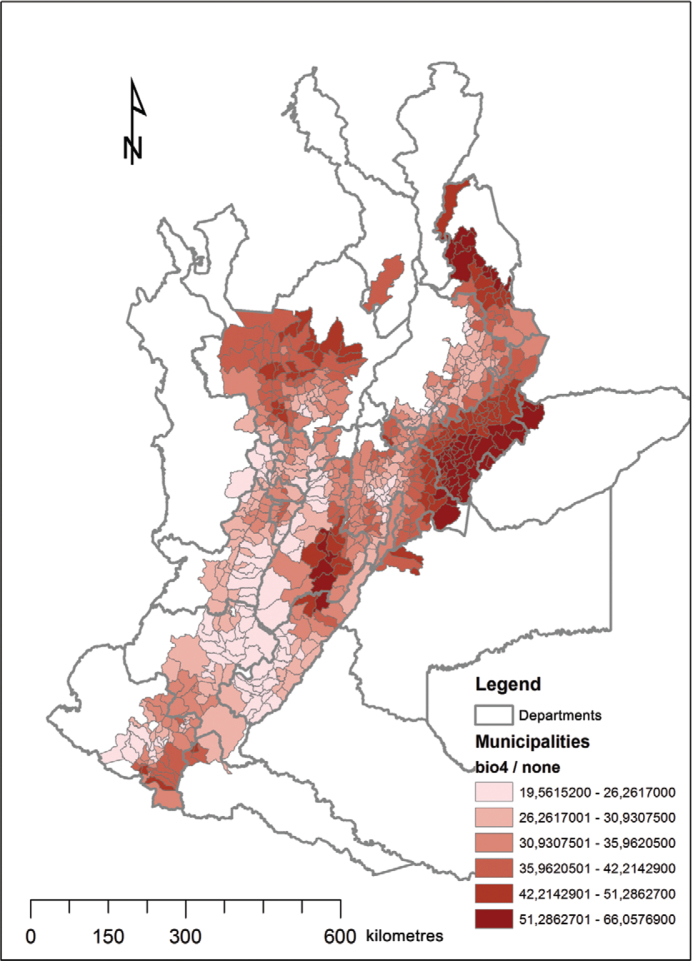
Map of temperature seasonality (standard deviation *100) - Bio4 - for Andean region of Colombia.

## Supplementary Data

Bioclimatic variables as on the Bioclim website (http://www.worldclim.org/bioclim)

BIO1 = Annual Mean Temperature

BIO2 = Mean Diurnal Range [Mean of monthly (max temp - min temp)]

BIO3 = Isothermality (BIO2/BIO7) (*100)

BIO4 = Temperature Seasonality (standard deviation *100)

BIO5 = Max Temperature of Warmest Month

BIO6 = Min Temperature of Coldest Month

BIO7 = Temperature Annual Range (BIO5-BIO6)

BIO8 = Mean Temperature of Wettest Quarter

BIO9 = Mean Temperature of Driest Quarter

BIO10 = Mean Temperature of Warmest Quarter

BIO11 = Mean Temperature of Coldest Quarter

BIO12 = Annual Precipitation

BIO13 = Precipitation of Wettest Month

BIO14 = Precipitation of Driest Month

BIO15 = Precipitation Seasonality (Coefficient of Variation)

BIO16 = Precipitation of Wettest Quarter

BIO17 = Precipitation of Driest Quarter

BIO18 = Precipitation of Warmest Quarter

BIO19 = Precipitation of Coldest Quarter

*Equation of the model (spatio-temporal statistical model)* - Assuming that the occurrence of cases of cutaneous leishmaniasis (*Y*_*it*_) in a municipality of *N*_*i*_ inhabitants follows a Poisson mixture distribution with mean *μ*_*it*_ (*μ*_*it*_ *= Y*_*it*_ */ N*_*i*_), the random-effects Poisson model used is:

Where:

*Y*_*it*_ is the number of cases of cutaneous leishmaniasis in the municipality *i* and year *t*

*µ*_*i*_ is the incidence rate of cutaneous leishmaniasis in the municipality *i* and year *t*

*N*_*i*_ is the number of inhabitants in the municipality *i*

*a*_0_ is the intercept

*a*_*t*_ is the parameter capturing the time (year) from 2000 to 2009, with 2004 as the reference level

*x*_*i*_ are the 31 independent variables (land use, temperature, rainfall, elevation and population density) with regression coefficient *a*_*ij*_

*a*_*ij*_ is the parameter capturing incidence effect in the municipality *i* and independent variable *j*

*b*_*i*_ are spatial random effects with conditional autoregressive (CAR) structure (Thomas et al. 2004)

*h*_*i*_ is the unstructured component of the random effect in the area *i*

**TABLE:** The matrix of spearman correlations (r) of climate variables

Variables	*ρ*	BIO1	BIO2	BIO3	BIO4	BIO5	BIO6	BIO7	BIO8	BIO9	BIO10	BIO11	BIO12	BIO13	BIO14	BIO15	BIO16	BIO17	BIO18	BIO19	Elev
BIO1	*ρ*	1,000	,502^**^	,402^**^	-,041	,992^**^	,992^**^	,129^**^	,999^**^	,999^**^	,999^**^	,999^**^	,400^**^	,420^**^	,348^**^	-,045	,380^**^	,368^**^	,145^**^	,451^**^	-,991^**^
	r-value		,000	,000	,277	0,000	0,000	,001	0,000	0,000	0,000	0,000	,000	,000	,000	,225	,000	,000	,000	,000	0,000
BIO2	*ρ*	,502^**^	1,000	,046	,011	,587^**^	,419^**^	,773^**^	,511^**^	,503^**^	,505^**^	,504^**^	,068	,103^**^	,014	,115^**^	,064	,015	-,149^**^	,056	-,478^**^
	r-value	,000		,217	,770	,000	,000	,000	,000	,000	,000	,000	,068	,006	,703	,002	,089	,688	,000	,137	,000
BIO3	*ρ*	,402^**^	,046	1,000	-,634^**^	,339^**^	,469^**^	-,559^**^	,399^**^	,402^**^	,382^**^	,415^**^	,384^**^	,258^**^	,711^**^	-,679^**^	,263^**^	,657^**^	,405^**^	,330^**^	-,403^**^
	r-value	,000	,217		,000	,000	,000	,000	,000	,000	,000	,000	,000	,000	,000	,000	,000	,000	,000	,000	,000
BIO4	*ρ*	-,041	,011	-,634^**^	1,000	-,001	-,080^*^	,388^**^	-,039	-,040	-,014	-,069	-,144^**^	-,042	-,463^**^	,541^**^	-,047	-,402^**^	-,258^**^	-,099^**^	,066
	r-value	,277	,770	,000		,988	,033	,000	,294	,289	,710	,064	,000	,267	,000	,000	,206	,000	,000	,008	,080
BIO5	*ρ*	,992^**^	,587^**^	,339^**^	-,001	1,000	,972^**^	,237^**^	,991^**^	,992^**^	,993^**^	,991^**^	,386^**^	,417^**^	,302^**^	,004	,375^**^	,327^**^	,105^**^	,442^**^	-,980^**^
	r-value	0,000	,000	,000	,988		0,000	,000	0,000	0,000	0,000	0,000	,000	,000	,000	,917	,000	,000	,005	,000	0,000
BIO6	*ρ*	,992^**^	,419^**^	,469^**^	-,080^*^	,972^**^	1,000	,020	,989^**^	,992^**^	,990^**^	,992^**^	,416^**^	,425^**^	,390^**^	-,097^**^	,389^**^	,408^**^	,167^**^	,474^**^	-,984^**^
	r-value	0,000	,000	,000	,033	0,000		,597	0,000	0,000	0,000	0,000	,000	,000	,000	,009	,000	,000	,000	,000	0,000
BIO7	*ρ*	,129^**^	,773^**^	-,559^**^	,388^**^	,237^**^	,020	1,000	,138^**^	,130^**^	,142^**^	,122^**^	-,204^**^	-,101^**^	-,440^**^	,501^**^	-,133^**^	-,409^**^	-,384^**^	-,174^**^	-,107^**^
	r-value	,001	,000	,000	,000	,000	,597		,000	,000	,000	,001	,000	,007	,000	,000	,000	,000	,000	,000	,004
BIO8	*ρ*	,999^**^	,511^**^	,399^**^	-,039	,991^**^	,989^**^	,138^**^	1,000	,997^**^	,998^**^	,998^**^	,391^**^	,411^**^	,349^**^	-,047	,369^**^	,367^**^	,146^**^	,431^**^	-,990^**^
	r-value	0,000	,000	,000	,294	0,000	0,000	,000		0,000	0,000	0,000	,000	,000	,000	,214	,000	,000	,000	,000	0,000
BIO9	*ρ*	,999^**^	,503^**^	,402^**^	-,040	,992^**^	,992^**^	,130^**^	,997^**^	1,000	,999^**^	,998^**^	,405^**^	,426^**^	,347^**^	-,045	,387^**^	,369^**^	,137^**^	,466^**^	-,989^**^
	r-value	0,000	,000	,000	,289	0,000	0,000	,000	0,000		0,000	0,000	,000	,000	,000	,228	,000	,000	,000	,000	0,000
BIO10	*ρ*	,999^**^	,505^**^	,382^**^	-,014	,993^**^	,990^**^	,142^**^	,998^**^	,999^**^	1,000	,998^**^	,398^**^	,422^**^	,331^**^	-,026	,382^**^	,354^**^	,138^**^	,451^**^	-,989^**^
	r-value	0,000	,000	,000	,710	0,000	0,000	,000	0,000	0,000		0,000	,000	,000	,000	,481	,000	,000	,000	,000	0,000
BIO11	*ρ*	,999^**^	,504^**^	,415^**^	-,069	,991^**^	,992^**^	,122^**^	,998^**^	,998^**^	,998^**^	1,000	,401^**^	,418^**^	,359^**^	-,060	,379^**^	,378^**^	,149^**^	,451^**^	-,990^**^
	r-value	0,000	,000	,000	,064	0,000	0,000	,001	0,000	0,000	0,000		,000	,000	,000	,110	,000	,000	,000	,000	0,000
BIO12	*ρ*	,400^**^	,068	,384^**^	-,144^**^	,386^**^	,416^**^	-,204^**^	,391^**^	,405^**^	,398^**^	,401^**^	1,000	,963^**^	,741^**^	-,282^**^	,972^**^	,819^**^	,830^**^	,903^**^	-,392^**^
	r-value	,000	,068	,000	,000	,000	,000	,000	,000	,000	,000	,000		0,000	,000	,000	0,000	,000	,000	,000	,000
BIO13	*ρ*	,420^**^	,103^**^	,258^**^	-,042	,417^**^	,425^**^	-,101^**^	,411^**^	,426^**^	,422^**^	,418^**^	,963^**^	1,000	,604^**^	-,066	,990^**^	,697^**^	,757^**^	,897^**^	-,413^**^
	r-value	,000	,006	,000	,267	,000	,000	,007	,000	,000	,000	,000	0,000		,000	,077	0,000	,000	,000	,000	,000
BIO14	*ρ*	,348^**^	,014	,711^**^	-,463^**^	,302^**^	,390^**^	-,440^**^	,349^**^	,347^**^	,331^**^	,359^**^	,741^**^	,604^**^	1,000	-,758^**^	,608^**^	,982^**^	,768^**^	,598^**^	-,358^**^
	r-value	,000	,703	,000	,000	,000	,000	,000	,000	,000	,000	,000	,000	,000		,000	,000	0,000	,000	,000	,000
BIO15	*ρ*	-,045	,115^**^	-,679^**^	,541^**^	,004	-,097^**^	,501^**^	-,047	-,045	-,026	-,060	-,282^**^	-,066	-,758^**^	1,000	-,099^**^	-,690^**^	-,423^**^	-,197^**^	,049
	r-value	,225	,002	,000	,000	,917	,009	,000	,214	,228	,481	,110	,000	,077	,000		,008	,000	,000	,000	,189
BIO16	*ρ*	,380^**^	,064	,263^**^	-,047	,375^**^	,389^**^	-,133^**^	,369^**^	,387^**^	,382^**^	,379^**^	,972^**^	,990^**^	,608^**^	-,099^**^	1,000	,696^**^	,758^**^	,914^**^	-,373^**^
	r-value	,000	,089	,000	,206	,000	,000	,000	,000	,000	,000	,000	0,000	0,000	,000	,008		,000	,000	,000	,000
BIO17	*ρ*	,368^**^	,015	,657^**^	-,402^**^	,327^**^	,408^**^	-,409^**^	,367^**^	,369^**^	,354^**^	,378^**^	,819^**^	,697^**^	,982^**^	-,690^**^	,696^**^	1,000	,813^**^	,684^**^	-,375^**^
	r-value	,000	,688	,000	,000	,000	,000	,000	,000	,000	,000	,000	,000	,000	0,000	,000	,000		,000	,000	,000
BIO18	*ρ*	,145^**^	-,149^**^	,405^**^	-,258^**^	,105^**^	,167^**^	-,384^**^	,146^**^	,137^**^	,138^**^	,149^**^	,830^**^	,757^**^	,768^**^	-,423^**^	,758^**^	,813^**^	1,000	,624^**^	-,145^**^
	r-value	,000	,000	,000	,000	,005	,000	,000	,000	,000	,000	,000	,000	,000	,000	,000	,000	,000		,000	,000
BIO19	*ρ*	,451^**^	,056	,330^**^	-,099^**^	,442^**^	,474^**^	-,174^**^	,431^**^	,466^**^	,451^**^	,451^**^	,903^**^	,897^**^	,598^**^	-,197^**^	,914^**^	,684^**^	,624^**^	1,000	-,439^**^
	r-value	,000	,137	,000	,008	,000	,000	,000	,000	,000	,000	,000	,000	,000	,000	,000	,000	,000	,000		,000
Elev	*ρ*	-,991^**^	-,478^**^	-,403^**^	,066	-,980^**^	-,984^**^	-,107^**^	-,990^**^	-,989^**^	-,989^**^	-,990^**^	-,392^**^	-,413^**^	-,358^**^	,049	-,373^**^	-,375^**^	-,145^**^	-,439^**^	1,000
	r-value	0,000	,000	,000	,080	0,000	0,000	,004	0,000	0,000	0,000	0,000	,000	,000	,000	,189	,000	,000	,000	,000	

*: the correlation is significant at the 0.05 level (bilateral); **: the correlation is significant at the 0.01 level (bilateral).

BIO1: Annual Mean Temperature

BIO2: Mean Diurnal Range [Mean of monthly (max temp - min temp)]

BIO3: Isothermality (BIO2/BIO7) (* 100)

BIO4: Temperature Seasonality (standard deviation *100)

BIO5: Max Temperature of Warmest Month

BIO6: Min Temperature of Coldest Month

BIO7: Temperature Annual Range (BIO5-BIO6)

BIO8: Mean Temperature of Wettest Quarter

BIO9: Mean Temperature of Driest Quarter

BIO10: Mean Temperature of Warmest Quarter

BIO11: Mean Temperature of Coldest Quarter

BIO12: Annual Precipitation

BIO13: Precipitation of Wettest Month

BIO14: Precipitation of Driest Month

BIO15: Precipitation Seasonality (Coefficient of Variation)

BIO16: Precipitation of Wettest Quarter

BIO17: Precipitation of Driest Quarter

BIO18: Precipitation of Warmest Quarter

BIO19: Precipitation of Coldest Quarter

Elev: Elevation
